# Pathological complete response due to a prolonged time interval between preoperative chemoradiation and surgery in locally advanced rectal cancer: analysis from the German StuDoQ|Rectalcarcinoma registry

**DOI:** 10.1186/s12885-020-6538-8

**Published:** 2020-01-20

**Authors:** Sven Lichthardt, Johanna Wagner, Stefan Löb, Niels Matthes, Caroline Kastner, Friedrich Anger, Christoph-Thomas Germer, Armin Wiegering

**Affiliations:** 1 Department of General, Visceral, Transplant, Vascular and Pediatric Surgery, University Hospital, Oberduerrbacherstr. 6, 97080 Wuerzburg, Germany; 2 University of Wuerzburg, Comprehensive Cancer Center Mainfranken, Wuerzburg, Germany; 3 Department of Biochemistry and Molecular Biology, Biocenter, University of Wuerzburg, Wuerzburg, Germany

**Keywords:** Rectal cancer, Surgery, Radiochemotherapy, Time interval

## Abstract

**Background:**

Preoperative chemoradiotherapy is the recommended standard of care for patients with local advanced rectal cancer. However, it remains unclear, whether a prolonged time interval to surgery results in an increased perioperative morbidity, reduced TME quality or better pathological response. Aim of this study was to determine the time interval for best pathological response and perioperative outcome compared to current recommended interval of 6 to 8 weeks.

**Methods:**

This is a retrospective analysis of the German StuDoQ|Rectalcarcinoma registry. Patients were grouped for the time intervals of “less than 6 weeks”, “6 to 8 weeks”, “8 to 10 weeks” and “more than 10 weeks”. Primary endpoint was pathological response, secondary endpoint TME quality and complications according to Clavien-Dindo classification.

**Results:**

Due to our inclusion criteria (preoperative chemoradiation, surgery in curative intention, M0), 1.809 of 9.560 patients were suitable for analysis. We observed a trend for increased rates of pathological complete response (pCR: ypT0ypN0) and pathological good response (pGR: ypT0-1ypN0) for groups with a prolonged time interval which was not significant. Ultimately, it led to a steady state of pCR (16.5%) and pGR (22.6%) in “8 to 10” and “more than 10” weeks. We were not able to observe any differences between the subgroups in perioperative morbidity, proportion of rectal extirpation (for cancer of the lower third) or difference in TME quality.

**Conclusion:**

A prolonged time interval between neoadjuvant chemoradiation can be performed, as the rate of pCR seems to be increased without influencing perioperative morbidity.

## Background

The most common malignant disease of the gastrointestinal tract is colorectal cancer (CRC) with about 1.3 million new cases each year worldwide [1, 2]. Rectal carcinoma (RC) – especially locally advanced rectal cancer (LARC) – is treated as an independent disease because of its anatomically proximity to the sphincter apparatus, a high local recurrence rate and different metastatic behavior [3]. This led to the development of different multimodal treatment strategies for LARC (UICC-stage II and III). A preoperative radiation or combined chemoradiotherapy is recommended for carcinomas of the lower or middle rectum due to a better local control and reduced rate of extirpation [4–6]. The German guidelines for colorectal cancer recommend the oncological resection 6 to 8 weeks after completed preoperative chemoradiotherapy [7]. Recent studies have shown that a prolonged interval leads to higher rates of pathological complete response (pCR) and that this my even take longer than 16 weeks [8–11]. In addition, it was shown, that additional inclusion of chemotherapy cycles in the interval between radiochemotherapy and surgery enhance complete response rate without affecting surgical morbidity [12]. However, the results of Lefevre et al. suggest that an increased time interval leads to more severe postoperative complications and a worse quality of total mesorectal excision (TME) [13].

Aim of this study was to determine the optimal time interval between chemoradiotherapy and surgery in LARC with respect to the primary endpoint pCR. The secondary endpoints were tumor regression grade (TRG), quality of TME and postoperative complications (ileus, anastomotic leak, bleeding, sacral wound healing disorder) according to the Clavien-Dindo-classification [14].

## Methods

### The StuDoQ|Rectalcarcinoma registry

The German Society for General and Visceral Surgery (DGAV) created a central register (StuDoQ) to evaluate the quality of healthcare and risk factors for different benign and malignant diseases, including colorectal cancer. The StuDoQ|Rectalcarcinoma registry (www.dgav.de/studoq) is a prospective registry, which contains anonymized data of patients with rectal cancer treated in German hospitals. Data from the participating clinics was included in a pseudonymized form. The DGAV established the publication guidelines (https://www.dgav.de/studoq/datenschutzkonzept-und-publikationsrichtlinien.html), while the Society for Technology, Methods, and Infrastructure for Networked Medical Research (http://www.tmf-ev.de/) established the data safety concept and ethical approvement [15]. The registry contains 150 items regarding patient characteristics, tumor stage, pre and postoperative therapy and complications. We received the data of all patients with LARC from August 2009 until February 2017 with the number StuDoQ-2017-0002 for scientific analysis. Patients within the registry are supposed to be treated according to the German guidelines for rectal cancer (https://www.awmf.org/uploads/tx_szleitlinien/021-007OLk_S3_Kolorektales-Karzinom-KRK_2019-01.pdf), including radiotherapy with 50,4 Gy and 5Fu. All data providing hospital are listed in Additional file 1: Table S1. As this is a register containing perioperative data, no data to clinical endpoints like progression-free survival or overall survival are available.

### Statistical analysis

Extracted data was analyzed by SPSS version 24. We divided the patients into four subgroups according to the time interval between the end of preoperative chemoradiotherapy and the oncological resection (less than 6 weeks, 6 to 8 weeks, 8 to 10 weeks and more than 10 weeks). We calculated categorial variables as absolute count and subgroup-specific percentage, whereas scale variables are shown as range and median. The significance level was set at *p* < 0.05. We used the univariate variance analysis for continuous data and the Pearson Chi-square-test or Fisher’s exact test for categorical variables.

### Patients selection and endpoints

The patient’s selection was performed due to a readout of the StuDoQ|Rectalcarcinoma database according to inclusion and exclusion criteria. First, all cases were sorted out without an informed consent as well as invalid data (especially missing of postoperative tumor therapy and follow-up data). Tumor-specific inclusion criteria were an absence of distant metastasis and a histopathological approved ypUICC-stage. We only included patients with a regularly completed preoperative chemoradiation and documented date of last radiation followed by an elective surgery with oncological resection within 200 days. Of note, documentation of the last radiation date is not an obligate information to submit a valid dataset and by this often missing.

Primary endpoint of this study was pathological complete response, which is defined as the histological proof of no more vital, residual tumor cells, neither in the resected rectum (ypT0) nor in the lymph nodes (ypN0). Secondary endpoints were tumor regression grade (TRG), quality of TME and common postoperative complications (ileus, anastomotic leak, bleeding, sacral wound healing disorder) according to the Clavien-Dindo-classification. TRG was defined according to Dworak’s classification [16]. 0: no tumor regression; 1: dominant tumor mass with obvious fibrosis and/or vasculopathy; 2: dominantly fibrotic changes with few tumor cells or groups (easy to find); 3: very few (difficult to find microscopically) tumor cells in fibrotic tissue with or without mucous substance; 4: no tumor cells, only fibrotic mass (total regression or response). The quality of TME was graded according to the M.E.R.C.U.R.Y.-classfication [17, 18] system into 3 grades: complete, nearly complete and incomplete. Histopathological examination is based on the mesorectal integrity, the existence of defects, the conical conformation of the excised tumor specimen and the regularity of the circumferential resection margin (CRM).

## Results

### Patients selection

**Fig. 1:**
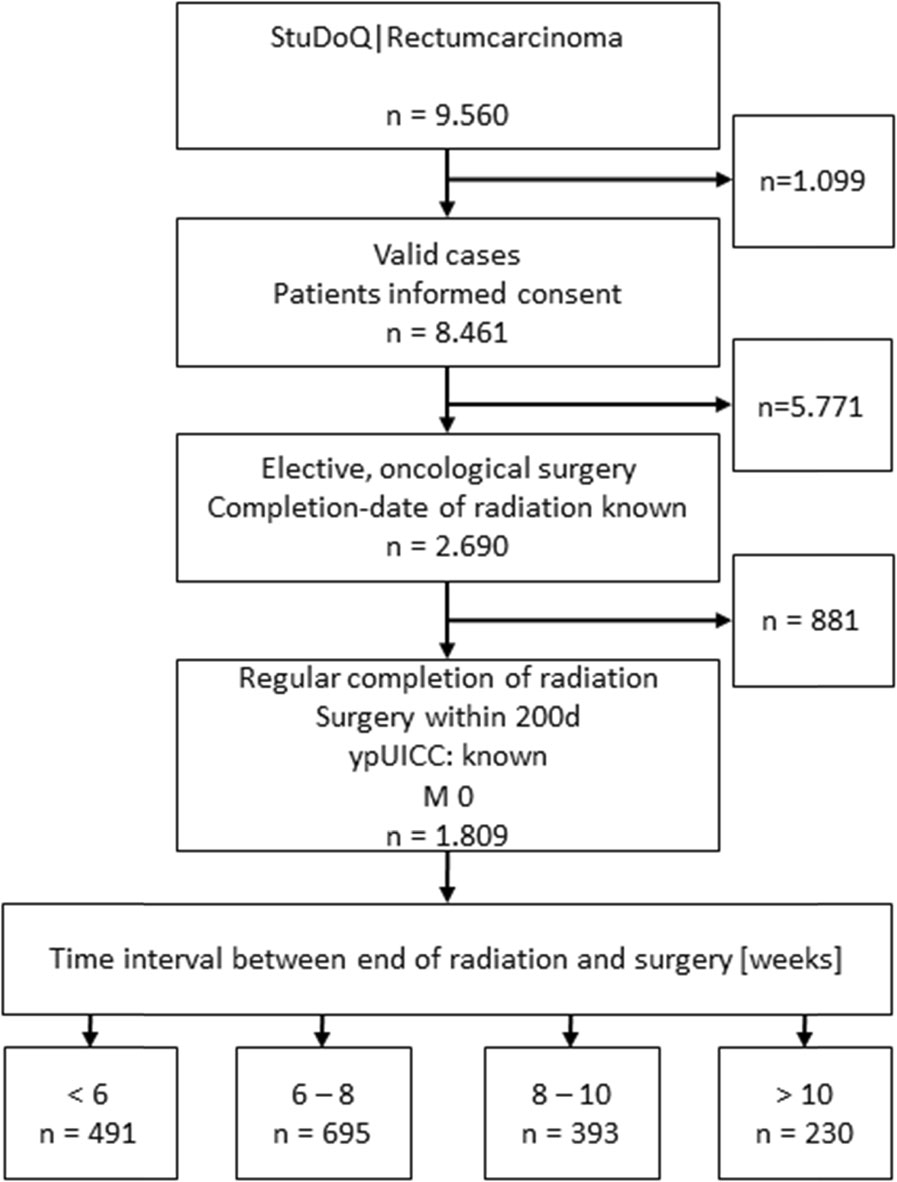
Flowchart of patients included

9.560 patients with the primary diagnosis of rectum carcinoma were registered in the StuDoQ-registry in April 2017 (Fig. 1). 1.099 patients were excluded due to invalid data assessment or a missing signed patient’s consent. For further analysis, only patients with LARC who undergo preoperative long-term radiochemotherapy with a known date of the beginning, end and completion of the preoperative chemoradiation were selected. Furthermore, only patients undergoing elective surgery had taken place within 200 days after completion of the preoperative therapy were selected. We also excluded patients with distant metastasis at primary diagnosis. In total, 1.809 patients were included and divided into 4 subgroups: “less than 6 weeks” (*n* = 491), “6 to 8 weeks” (as the recommended interval; *n* = 695), “8 to 10 weeks” (*n* = 393) and “more than 10 weeks” (*n* = 230) after the completion of the preoperative therapy.

### Patient characteristics

Table 1 shows the patient characteristics of the included cohort. More than half of the patients were male (67.2%). The median age was 66 years at the time of diagnosis and the median BMI was 25.7 kg/m^2^. Most patients were in a good medical condition according to the ASA physical status classification system (ASA: American Society of Anesthesiologists). Patients with an ASA-score III were – while not significantly – more likely to get surgery earlier. There were no significant differences between the four subgroups concerning common comorbidities, such as diabetes mellitus, coronary heart disease or chronic heart failure. The location of the primary tumor was in 95.5% in the lower or middle rectum third. 71 patients (3.9%) had their primary tumor location more than 12 cm from the anal verge.

**Table 1:**
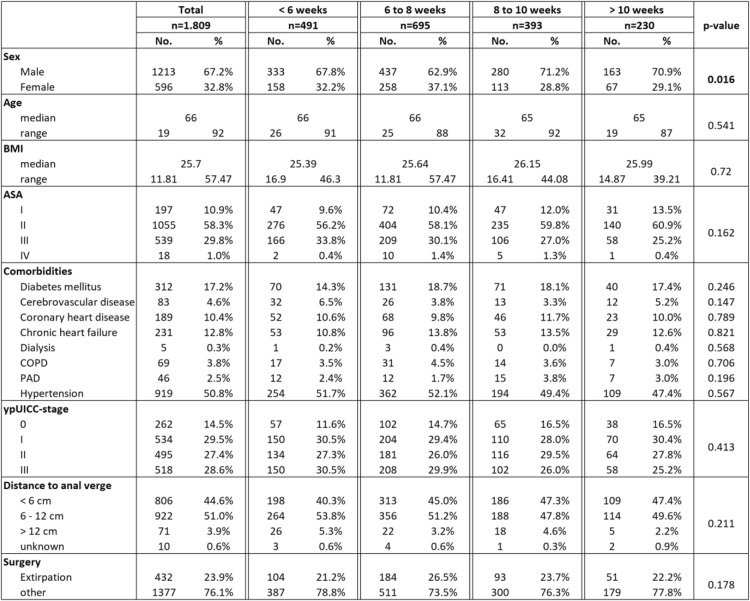
Patients characteristics

### Pathological response and tumor regression

**Fig. 2:**
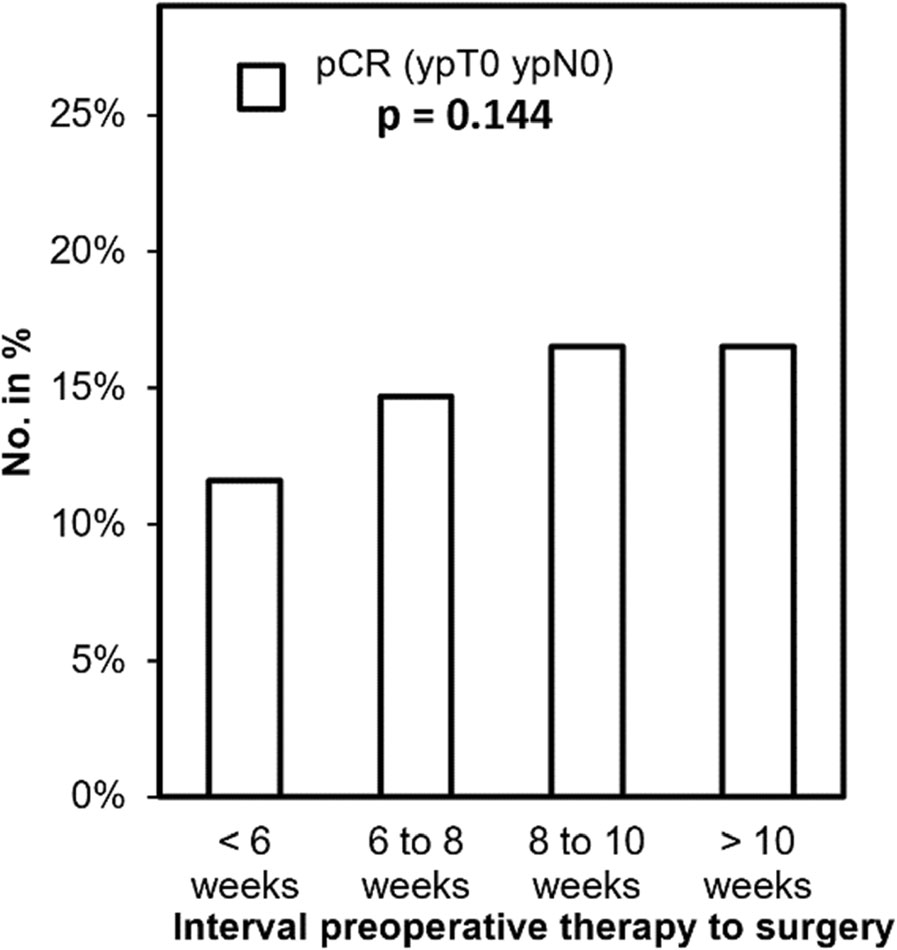
Pathological complete (pCR) response according to the time interval between preoperative therapy and surgery

**Fig. 3:**
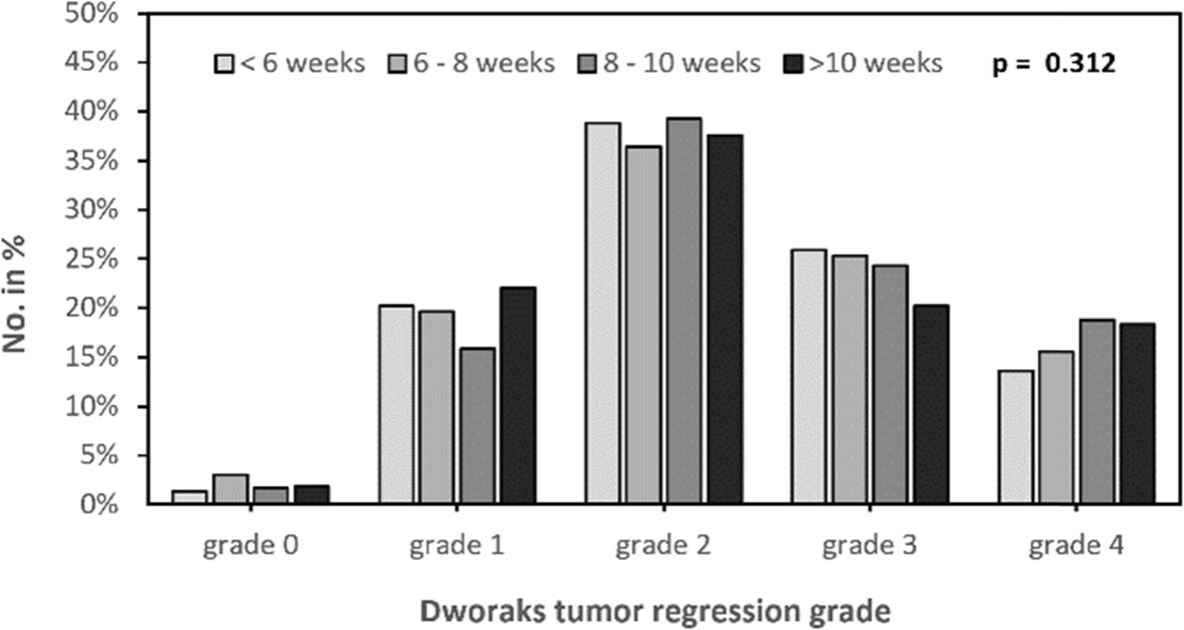
Tumor regression grade according to the time interval between end of radiation and surgery

pCR was defined as primary endpoint in our study. Complete pathological response (pCR) is defined by missing vital tumor cells in the definitive histopathological staining of tumor specimen, neither in the primary tumor nor the draining lymph nodes (ypT0ypN0). Figure 2 shows the rate of pCR according to the time interval between preoperative therapy and radical resection. There was no significant difference in the percentage of pCR of pretreated tumor specimen with regard to different time intervals (*p* = 0.144). This accounted for the comparison of all subgroups as well as for the comparison of each subgroup with the defined standard (control group “6 to 8 weeks”; *p* > 0.05). However, the lowest pCR-rates were found in the “< 6 weeks” group (11.6%). Furthermore, we detected a trend towards higher pCR in the groups with a longer interval between end of neoadjuvant treatment and surgical resection. Interestingly, the rate of pCR seems to undergo a regression leading to a steady state (> 8 weeks: 16.5%). As the time interval of 6 to 8 weeks is the recommended standard of treatment, we performed subgroup analysis comparing this group to other time intervals. We did not detect any significant differences. Furthermore, tumor regression grade (TRG) according to the tumor regression system of Dworak et al. [16] was investigated. TRG was documented for 1632 out of 1809 patients. To verify the results of the documented TRG we compared it to the pCR, which showed a significant dependency (*p* < 0.001). The TRG is shown according to the time interval to neoadjuvant chemoradiation in Fig. 3. Most patients with TRG 4 received surgery in more than 8 weeks (8 to 10 weeks: 18.8%; > 10 weeks: 18.3%) whereas earlier surgery resulted in lower tumor regression (TRG 4: < 6 weeks: 13.6%; 6 to 8 weeks: 15.6%). No significant differences between the groups have been noticed (*p* = 0.312).

### TME quality

**Fig. 4:**
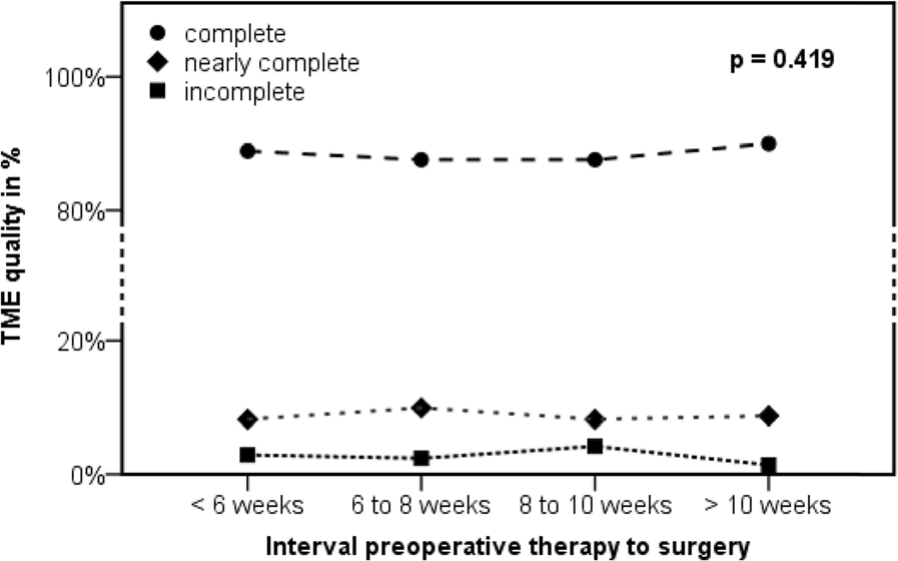
Quality of the total mesorectal excision (TME) according to the time interval between end of radiation and surgery

The TME quality did not show significant differences between the four subgroups (*p* = 0.419, Fig. 4). Complete TME was reached in 87.6% in the subgroups “6 to 8 weeks” and “8 to 10 weeks”. The rate of complete TME was slightly higher in the group “< 6 weeks” (88.9%) and “> 10 weeks” (90.0%). Nearly complete TME ranged from 8.2 to 9.9%, independent of length of the time interval. The subgroup “8 to 10 weeks” showed the highest rate of incomplete TME (4.2%), while the other subgroups ranged between 1.4 and 2.9% (1.4% “> 10 weeks”; 2.4% “6 to 8 weeks”; 2.9% “< 6 weeks”).

### Complication rate

**Fig. 5:**
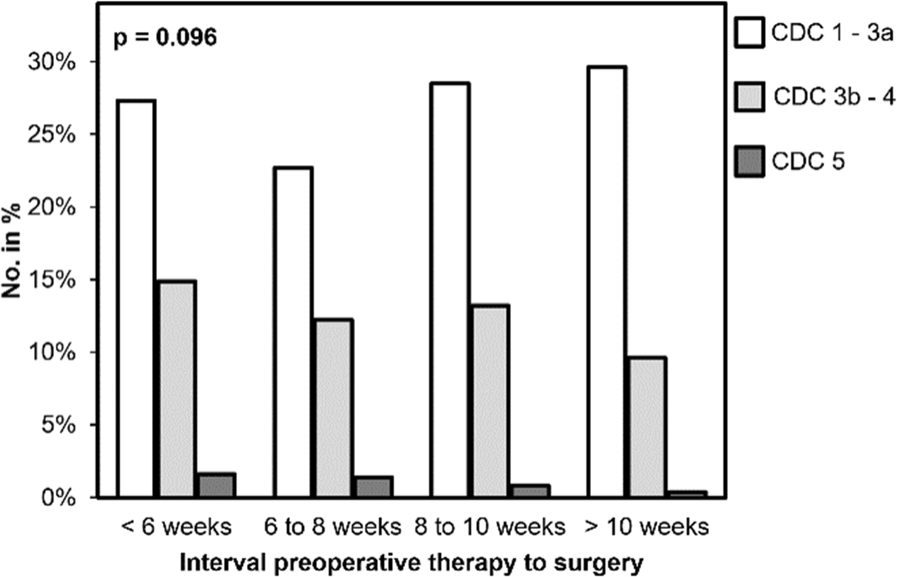
Clavien-Dindo classification (CDC) according to the time interval

Postoperative complications graded by the Clavien-Dindo classification (CDC) were subsumed to the following subgroups: CDC 1 to 3a (defined as minor complications), 3b to 4 (defined as major complications) and 5 (defined as fatal complications). There were no significant differences in the rate of postoperative complications depending on the time interval, neither for the above-mentioned CDC-subgroups (*p* = 0.096) nor the ungrouped CDC (*p* = 0.106). Minor complications (CDC 1 – 3a) were more common in the groups with a prolonged time interval (29.6% for “> 10 weeks” and 28.5% for “8 to 10 weeks”) while no significant differences between the time interval subgroups has been noticed (*p* = 0.07). In comparison with the control-group (“6 to 8 weeks”: CDC 1 – 3a: 22.7%) minor complications appeared significantly less frequent compared to longer time intervals (“8 to 10 weeks”: *p* = 0.034; “> 10 weeks”: *p* = 0.037). On the flip side we observed a trend towards major and fatal complications in patients resected early after completion of neoadjuvant treatment. Major complications (CDC 3b – 4) decreased from 14.9% in the group with the shortest time interval (“< 6 weeks”) to 9.6% to the group with the longest time interval (“> 10 weeks”). We found a comparable decrease in incidence for fatal complications from 1.6% (“< 6 weeks) to 0.4% (“> 10 weeks”). However, there was no significant difference between the subgroups or compared to the “6 to 8 weeks”-subgroup, neither for major nor for fatal complications. Figure 5 summarizes these results.

Furthermore, we examined the most common postoperative complications in colorectal surgery separately. The rates of postoperative bleeding in general as well as postoperative bleeding requiring transfusion, were less than 2.5% in all subgroups. There were no significant differences in our performed subgroup analysis (*p* > 0.5).

**Fig. 6:**
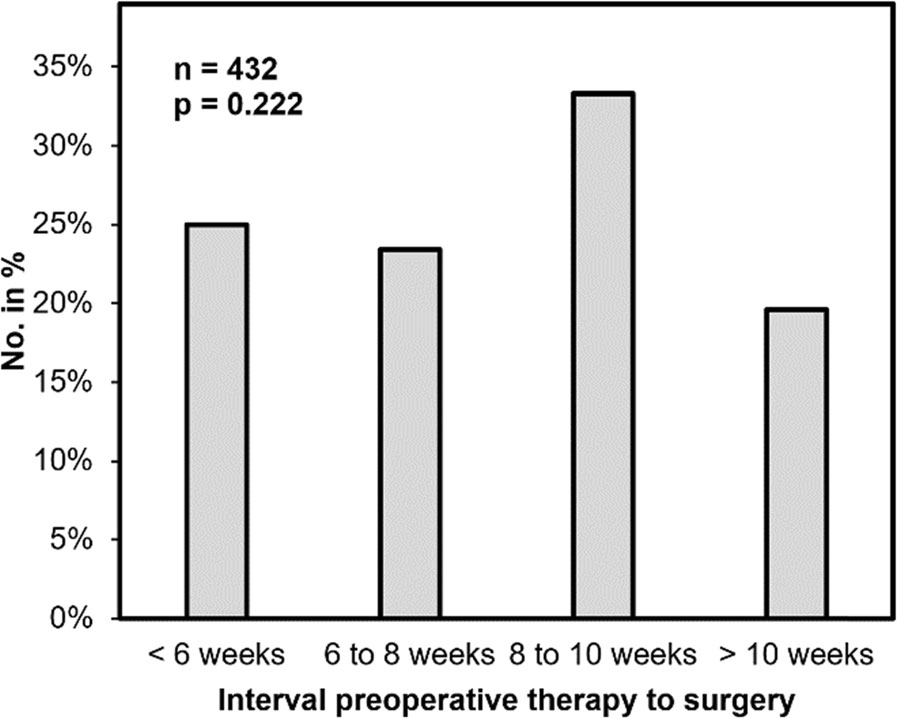
Sacral wound healing disorder in patients with rectal extirpation (*n* = 432) according to the time interval between preoperative therapy and surgery

The portion of postoperative ileus was highest in the subgroups “< 6 weeks” (4.9%) and “8 to 10 weeks” (5.1%), whereas it only occurred in 2.7% (“6 to 8 weeks”) and 2.2% (“> 10 weeks”) in the other two subgroups. The differences showed a trend but no significance (*p* = 0.064). A comparison to the “6 to 8 weeks” did not show any significant difference. We observed sacral wound healing disorder in 110 of all 432 patients with rectal extirpation (25.5%). The subgroup analysis showed non-significant difference according to the time interval: 19.6% (“> 10 weeks”), 33.3% (“8 to 10 weeks”), 23.4% (“6 to 8 weeks”) and 25.0% (“< 6 weeks) with a *p*-value of 0.222 (Fig. 6). No continuous dependency to the time interval was observed.

**Fig. 7:**
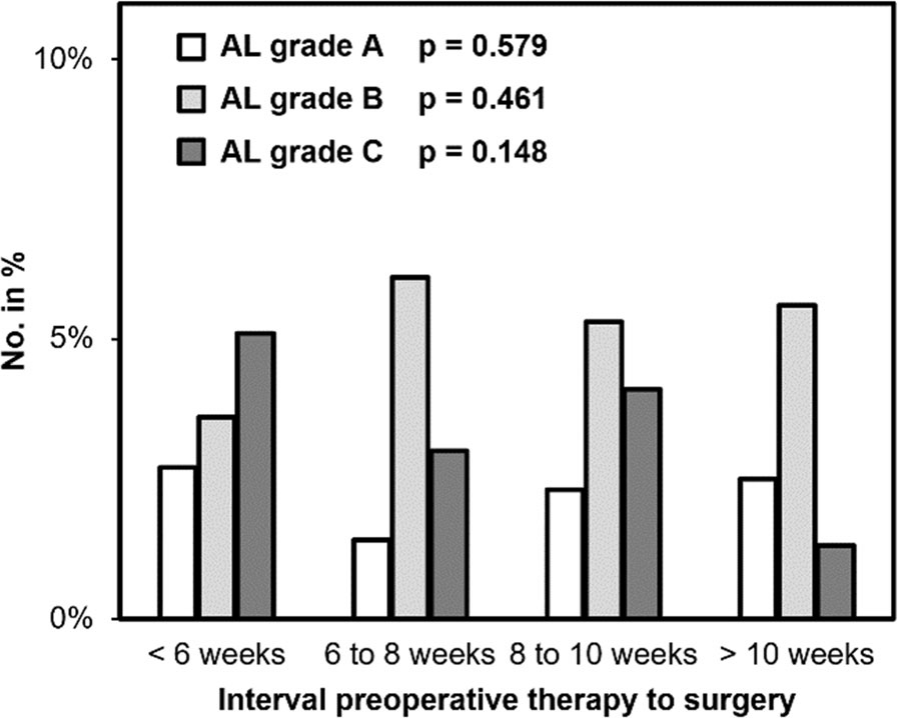
Anastomotic leakage in patients with rectal resection under stoma protection (*n* = 1187) according to the time interval between neoadjuvant chemoradiation and surgery

Finally, we investigated the rate of anastomotic leakage (AL) in 1187 patients after low anterior rectal resection with a primary anastomosis and a defunctioning ileostomy (Fig. 7). We found an overall anastomotic leakage in 10.8% of patients. AL grade A (no intervention needed) was lowest in the control group “6 to 8 weeks” (1.4%) and highest in the “< 6 weeks” group (2.7%). Only 3.6% of patients in the subgroup “< 6 weeks” and 5.3 to 6.1% of patients in the other subgroups developed an AL grade B (intervention without relaparotomy). The rate of AL grade C (relaparotomy needed) was highest in the shortest time interval with 5.6% decreasing to 1.2% in the longest time interval (“> 10 weeks”). We did not observe significant differences in the subgroups (*p* = 0.208).

## Discussion

In the provided analysis of a large cohort of 1.809 patients with rectal cancer undergoing preoperative therapy we found that the rate of pathological complete response (pCR), as well as the tumor regression grade (TRG) did not differ significant by prolonging the time interval between preoperative chemoradiation and surgery. Although our results could not show significant differences in pCR or TRG, there is a trend to higher rates of pCR and TRG with increase of the interval to surgery. This trend ends in a steady state of 16.5% by prolonging the time over 8 weeks. This might result in a better oncological outcome as there is evidence for a prolonged overall and disease-free survival for patients with pCR [19–21]. Furthermore, the results of the secondary endpoints suggest that a prolonged time interval does not affect the rate of postoperative complications, the rate of rectal extirpation or has an impact on TME quality.

Previous retrospective analysis due to the time interval of chemoradiation and surgery of rectal cancer have been made. All of them gained evidence for an increased rate of pCR by prolonging the time interval between neoadjuvant treatment and surgery without affecting perioperative morbidity [8–10, 22–25]. These studies analyzed patients from the United States, the Netherlands or Belgium, whereas the number of patients was partly low (e.g. 177 [22] or 356 [25]). Therefore, we conducted an analysis with a larger number of patients treated in Germany. Our results are in line with previous retrospective analysis.

Surprisingly, the results of the only multicenter, randomized, controlled trial published in 2016 by Lefévre and colleagues [13] displayed no affection of the pCR-rate in terms of time between neoadjuvant treatment and surgical resection, but a worsened local control and higher perioperative morbidity. In the meantime, the 3-year survival results of the GRECCAR-6 trial were published, showing that a prolonged time interval has no influence on oncological outcome of T3/T4 rectal cancer [26].

In light of the literature of retrospective analysis and our own data it could be suggested that a prolonged time interval over the recommended 6 to 8 weeks could result in higher rates of pCR and therefore better oncological outcome. As there is a harsh contrast to the only randomized controlled trial with the highest evidence, it remains unclear, why all database analysis seem to fail in the same direction.

Our results must be analyzed critically since this is a database analysis undergoing several limitations. The quality of analyzed data relies on the completeness and correctness of data provided by each individual hospital. A major problem falls to the invalid data as over a thousand patients had to be excluded, mostly because of missing data of postoperative follow-up and tumor therapy. In line with this we had to exclude nearly 2/3 of all patients due to missing end date of neoadjuvant therapy as this is a facultative parameter within the registry. Also, it remains unclear, why several decisions have been made, such as patients receiving a neoadjuvant treatment with cancer of the upper rectum or not receiving mesorectal excision. Furthermore, we can not draw conclusions, why over 60% of patients with locally advanced rectal cancer did not receive the recommended therapy (german S3-guidline for rectal cancer) within the 6 to 8 weeks interval. Moreover, no observation concerning disease-free and overall survival can be made. Nevertheless, they display real-world-data showing the practiced standard of treatment for patients with locally advanced rectal cancer in German hospitals.

## Conclusion

Our data suggest a prolonged time interval between end of chemoradiation and oncological resection in patients with locally advanced rectal cancer can be benefit for higher rates of pCR and TRG without increased perioperative morbidity. It still remains elusive if this we also lead to a higher overall survival rate.

## Supplementary information

**Additional file 1: Table S1.** All participating hospitals and surgical directors who contributed patient data to the StuDoQ|ColonCancer registry.

## Data Availability

The datasets generated and/or analysed during the current study are not publicly available due data safety protection guidelines of the DGAV StuDoQ Registry but are available from the corresponding author on reasonable request.

## Abbreviations

CRC: Colorectal cancer
LARC: Locally advanced rectal cancer
pCR: Pathological complete response
RC: Rectal carcinoma
TME: Total mesorectal excision
TRG: Tumor regression grade

## References

[1] Siegel RL (2017). Colorectal cancer statistics, 2017. CA Cancer J Clin.

[2] Torre LA (2015). Global cancer statistics, 2012. CA Cancer J Clin.

[3] Wiegering A (2014). Multimodal therapy in treatment of rectal cancer is associated with improved survival and reduced local recurrence - a retrospective analysis over two decades. BMC Cancer.

[4] Camma C (2000). Preoperative radiotherapy for resectable rectal cancer: a meta-analysis. JAMA.

[5] Sauer R (2004). Preoperative versus postoperative chemoradiotherapy for rectal cancer. N Engl J Med.

[6] Sauer R (2012). Preoperative versus postoperative chemoradiotherapy for locally advanced rectal cancer: results of the German CAO/ARO/AIO-94 randomized phase III trial after a median follow-up of 11 years. J Clin Oncol.

[7] Schmiegel W (2017). Z Gastroenterol.

[8] Foster JD (2013). Timing of surgery after long-course neoadjuvant chemoradiotherapy for rectal cancer: a systematic review of the literature. Dis Colon Rectum.

[9] Petrelli F (2016). Increasing the interval between Neoadjuvant Chemoradiotherapy and surgery in rectal Cancer: a meta-analysis of published studies. Ann Surg.

[10] Wang XJ (2016). Effect of interval between Neoadjuvant Chemoradiotherapy and surgery on oncological outcome for rectal Cancer: a systematic review and meta-analysis. Gastroenterol Res Pract.

[11] Habr-Gama Angelita, São Julião Guilherme P., Fernandez Laura M., Vailati Bruna B., Andrade Andres, Araújo Sérgio E. A., Gama-Rodrigues Joaquim, Perez Rodrigo O. (2019). Achieving a Complete Clinical Response After Neoadjuvant Chemoradiation That Does Not Require Surgical Resection. Diseases of the Colon & Rectum.

[12] Garcia-Aguilar J (2015). Effect of adding mFOLFOX6 after neoadjuvant chemoradiation in locally advanced rectal cancer: a multicentre, phase 2 trial. Lancet Oncol.

[13] Lefevre JH (2016). Effect of interval (7 or 11 weeks) between Neoadjuvant Radiochemotherapy and surgery on complete pathologic response in rectal Cancer: a multicenter, randomized, controlled trial (GRECCAR-6). J Clin Oncol.

[14] Dindo D, Demartines N, Clavien PA (2004). Classification of surgical complications: a new proposal with evaluation in a cohort of 6336 patients and results of a survey. Ann Surg.

[15] Wellner UF (2017). Laparoscopic versus open distal pancreatectomy-a propensity score-matched analysis from the German StuDoQ|pancreas registry. Int J Color Dis.

[16] Dworak O, Keilholz L, Hoffmann A (1997). Pathological features of rectal cancer after preoperative radiochemotherapy. Int J Color Dis.

[17] Herzog T (2010). TME quality in rectal cancer surgery. Eur J Med Res.

[18] Bartram C, Brown G (2002). Endorectal ultrasound and magnetic resonance imaging in rectal cancer staging. Gastroenterol Clin N Am.

[19] Maas M (2010). Long-term outcome in patients with a pathological complete response after chemoradiation for rectal cancer: a pooled analysis of individual patient data. Lancet Oncol.

[20] Martin ST, Heneghan HM, Winter DC (2012). Systematic review and meta-analysis of outcomes following pathological complete response to neoadjuvant chemoradiotherapy for rectal cancer. Br J Surg.

[21] Zorcolo L (2012). Complete pathologic response after combined modality treatment for rectal cancer and long-term survival: a meta-analysis. Ann Surg Oncol.

[22] de Campos-Lobato LF (2011). Neoadjuvant therapy for rectal cancer: the impact of longer interval between chemoradiation and surgery. J Gastrointest Surg.

[23] Probst CP (2015). Extended intervals after Neoadjuvant therapy in locally advanced rectal Cancer: the key to improved tumor response and potential organ preservation. J Am Coll Surg.

[24] Sloothaak DA (2013). Optimal time interval between neoadjuvant chemoradiotherapy and surgery for rectal cancer. Br J Surg.

[25] Wolthuis AM (2012). Impact of interval between neoadjuvant chemoradiotherapy and TME for locally advanced rectal cancer on pathologic response and oncologic outcome. Ann Surg Oncol.

[26] Lefevre JH (2019). Does a longer waiting period after Neoadjuvant radio-chemotherapy improve the oncological prognosis of rectal Cancer?: three Years' follow-up results of the Greccar-6 randomized multicenter trial. Ann Surg.

